# Evidence for Protein–Protein Interaction between Dopamine Receptors and the G Protein-Coupled Receptor 143

**DOI:** 10.3390/ijms22158328

**Published:** 2021-08-03

**Authors:** Beatriz Bueschbell, Prashiela Manga, Erika Penner, Anke C. Schiedel

**Affiliations:** 1 Center for Neuroscience and Cell Biology, University of Coimbra, 3004-504 Coimbra, Portugal; btrb@uni-bonn.de; 2 PhD Programme in Experimental Biology and Biomedicine, Institute for Interdisciplinary Research (IIIUC), University of Coimbra, Casa Costa Alemão, 3030-789 Coimbra, Portugal; 3 Ronald O. Perelman Department of Dermatology, Grossman School of Medicine, New York University, New York, NY 10016, USA; Prashiela.Manga@nyulangone.org; 4 Department of Pharmaceutical & Medicinal Chemistry, Pharmaceutical Institute, University of Bonn, D-53121 Bonn, Germany; erikapenner@hotmail.de

**Keywords:** dopamine receptors, Parkinson’s disease, GPR143, DRD_2_, DRD_3_

## Abstract

Protein-protein interactions between G protein-coupled receptors (GPCRs) can augment their functionality and increase the repertoire of signaling pathways they regulate. New therapeutics designed to modulate such interactions may allow for targeting of a specific GPCR activity, thus reducing potential for side effects. Dopamine receptor (DR) heteromers are promising candidates for targeted therapy of neurological conditions such as Parkinson’s disease since current treatments can have severe side effects. To facilitate development of such therapies, it is necessary to identify the various DR binding partners. We report here a new interaction partner for DRD_2_ and DRD_3_, the orphan receptor G protein-coupled receptor 143 (GPR143), an atypical GPCR that plays multiple roles in pigment cells and is expressed in several regions of the brain. We previously demonstrated that the DRD_2_/ DRD_3_ antagonist pimozide also modulates GPR143 activity. Using confocal microscopy and two FRET methods, we observed that the DRs and GPR143 colocalize and interact at intracellular membranes. Furthermore, co-expression of wildtype GPR143 resulted in a 57% and 67% decrease in DRD_2_ and DRD_3_ activity, respectively, as determined by β-Arrestin recruitment assay. GPR143-DR dimerization may negatively modulate DR activity by changing affinity for dopamine or delaying delivery of the DRs to the plasma membrane.

## 1. Introduction

G protein-coupled receptors (GPCRs) constitute the largest family of transmembrane proteins and are involved in almost all (patho)physiological processes. GPCRs can contribute directly to disease due to pathogenic mutations that modulate function or modify expression levels, but are also excellent therapeutic targets. Over 35% of all marketed drugs target GPCRs, rendering them the largest class of drug targets [1,2].

GPCRs were previously thought to function as monomers; however, it is now widely accepted that the receptors form highly dynamic, yet specific, functional homo- and/or heterodimers or even higher-order oligomers that can increase the number of roles a single protein can play [3,4]. Formation of heteromers can modulate the GPCR activity by influencing ligand recognition sites (e.g., changing or creating orthosteric and allosteric binding sites), G protein-coupling, and switching from G protein- to β-Arrestin-coupling [5]. The structural basis underlying dimerization and receptor modulation is not fully understood, and the role of dimers in pathological conditions such as asthma, cardiac failure, and neurological diseases has only been reported in recent years [3]. These interactions can thus provide novel opportunities for future drug development. 

Dopamine receptors (DRs, DRD_1_-DRD_5_) are a family of GPCRs that regulate numerous physiological functions including vision, cognitive function, and voluntary movement. They have been associated with pathological conditions and mental disorders such as Parkinson’s disease, schizophrenia, and nicotine addiction [6,7,8]. DR activity can be modulated by dimerization and/or oligomerization with other DRs as well as other GPCRs such as adenosine A_1_ receptors, glutamate *N*-methyl-d-aspartate receptors, or cannabinoid 1 receptors, which can further diversify and fine-tune their function [9,10,11,12,13,14]. For example, DRD_2_ forms hetero-dimers and high-order oligomers with adenosine 2A receptors (A_2A_AR) [11,15,16] that block activation of dopaminerigc transmission by A_2A_AR, as well as the modulation of mitogen-activated protein kinase (MAPK) responses [17,18]. DRD_3_-A_2A_AR-complexes display similar allosteric antagonistic receptor–receptor interactions [19,20,21].

We previously performed a chemical library screen to identify small molecules that modulate activity of the orphan receptor G protein-coupled receptor 143 (GPR143) and identified pimozide as an antagonist [22]. Pimozide is an oral antipsychotic that is also an antagonist of DRD_2_ and -D_3_ [23,24,25,26], we therefore hypothesized that, given the overlap in areas of expression in the eyes and brain [27,28], GPR143 could interact with these DRs. 

GPR143 is an atypical GPCR found primarily in pigment cells but is also expressed in some neurons [29]. In pigment cells, it is localized intracellularly at endolysosomes and melanosomes (specialized organelles in which the pigment melanin is synthesized) rather than the cell membrane where most other GPCRs function [30,31,32]. GPR143 mutations result in ocular albinism type 1 (OA1) [33], an X-linked recessive disorder that is characterized by visual anomalies including loss of stereoscopic vision due to misrouting of the optic fibers at the optic chiasm [34,35]. 

The precise role of GPR143 remains to be determined; however, modulation and translocation of other proteins may be a key feature of its function—for example, the melanocyte protein melanoma antigen recognized by T cells-1 (MART-1 ) has been shown to interact with GPR143, which serves as an escort/stabilizing protein [36]. Furthermore, GPR143 is co-immunoprecipitated with tubulin, suggesting a physical interaction with the cytoskeleton [37]. GPR143 has also been shown to physically interact with the heterotrimeric G-protein Gα_i3_ [38] and fine-tune activity of the vascular alpha-1B adrenergic receptor [39]. Given the propensity for GPR143 to interact with other proteins and our finding that the DRD_2_/-D_3_ antagonist pimozide also modulates GPR143 activity, we hypothesized that GPR143 could also interact with these DRs and modulate their activity.

## 2. Results

### 2.1. Fluorescence Resonance Energy Transfer 

We used fluorescence resonance energy transfer (FRET) in order to investigate GPR143-DR interactions. FRET analysis allows observation of interactions between two proteins localized within 10 nm of each other. 

**Figure 1:**
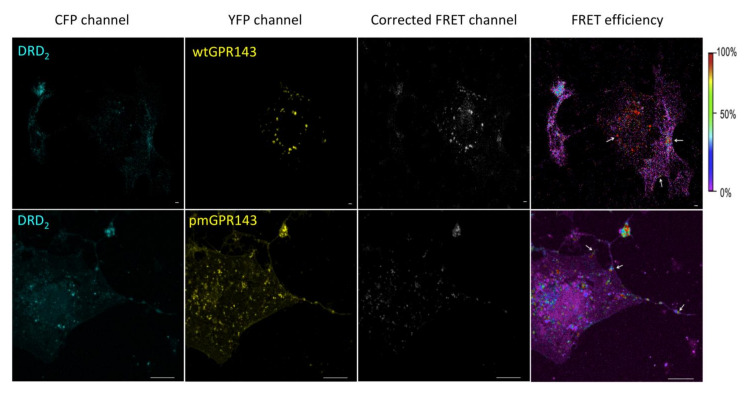
FRET of GPR143-YFP and DRD_2_-CFP in COS7 cells. The sensitized emission method was used to detect interaction between GPR143 (YFP channel) and DRD_2_ (CFP channel). FRET signal, corrected by CoA and CoB parameters, and FRET efficiency (color scale on the far right) are shown. White arrows indicate regions where FRET signal is localized. Controls are shown in Figure S2. Scale bar = 20 μm.

**Figure 2:**
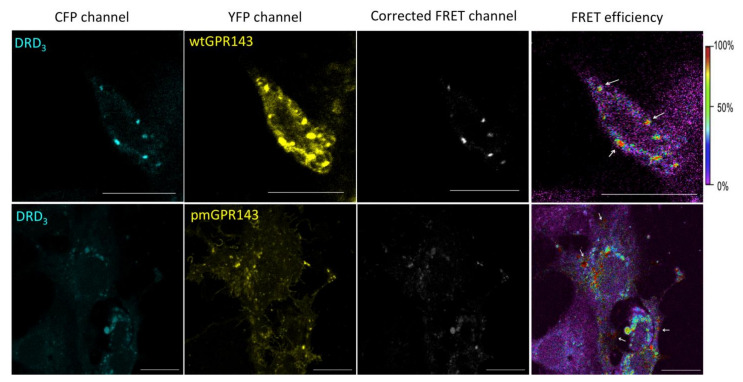
FRET of GPR143-YFP and DRD_3_-CFP in COS7 cells. A sensitized emission method was used to detect interaction between GPR143 (YFP channel) and DRD_3_ (CFP channel). FRET signal, corrected by CoA and CoB parameters, and FRET efficiency (color scale on the far right) are shown. White arrows indicate regions where the FRET signal is localized. Controls are shown in Figure S2. Scale bar = 20 μm.

We first used the sensitized emission method to evaluate FRET efficiency. If proteins are in close enough proximity for energy transfer to occur, donor fluorophore excitation (cyan fluorescent protein, CFP) leads to acceptor molecule emission (yellow fluorescent protein, YFP). Images of transfected cells were simultaneously acquired in all three channels (CFP, YFP, and FRET, Figure 1 and Figure 2). Correction parameters (CoA and CoB) were calculated by means of single transfected COS7s (Figure S1) and FRET efficiency values of each pixel in a pixel-to-pixel manner. This way, a FRET efficiency distribution across the picture was obtained, color-coded in a transition from purple to red (0 to 100 %; Figure 1 and Figure 2, right panels). A high FRET efficiency indicates an intense protein--protein interaction. 

In this study, we used two GPR143 expression plasmids, one encoding wildtype GPR143 (wtGPR143) that is usually found on melanosomes of melanocytes (pigment cells) and in late endosomal/lysosomal fractions in the intracellular space in non-menocytic cells [1,40,41,42,43]. The other encodes a mutant GPR143 that is fully functional but localizes to the plasma membrane (pmGPR143). Plasmids were generated as previously described [1]. 

In the sensitized emission FRET experiments, when wtGPR143 was co-expressed with DRD_2_, the FRET signal was observed in several vesicles around the perinuclear region, as well as near the plasma membrane with lower FRET efficiency (Figure 1, upper panels, white arrows). Similarly, when wtGPR143 was co-expressed with DRD_3_, the FRET signal was exclusively found in vesicles in the intracellular space around the perinuclear region (Figure 2, upper panels). pmGPR143 was found to interact with DRD_2_ and DRD_3_ in vesicles at or near the plasma membrane and spread at intracellular locations (Figure 1 and Figure 2, lower panels, white arrows). In control experiments with A_2A_AR, known to bind DRs, A_2A_AR-DRD_2_ and A_2A_AR-DRD_3_ interactions (up to 100% FRET efficiency) were mostly found at the plasma membrane (Figure S1, panels B and C). When comparing the double transfected with the single transfected COS7 cells (Figure S1, panel F and G), it was evident that co-transfection caused a change in the localization of the DRs. When the DRs were expressed alone, they showed a uniform distribution across the cell, while co-expression of wtGPR143 or pmGPR143 increased intracellular localization of the DRs. In contrast, co-expression of A_2A_AR or GPR18 did not cause a similar internal restriction of DRs (Figure S1, panels B-E). GPR18, a cannabinoid related orphan receptor [44], was used as negative control, since no interaction between GPR18 and DRs has been reported to date. When GPR18 was coexpressed with DRs, there was no evidence of interaction (Figure S1, panels D and E) and distribution within the cells did not differ from single transfected COS7 cells (Figure S1, panels F–I). 

**Figure 3:**
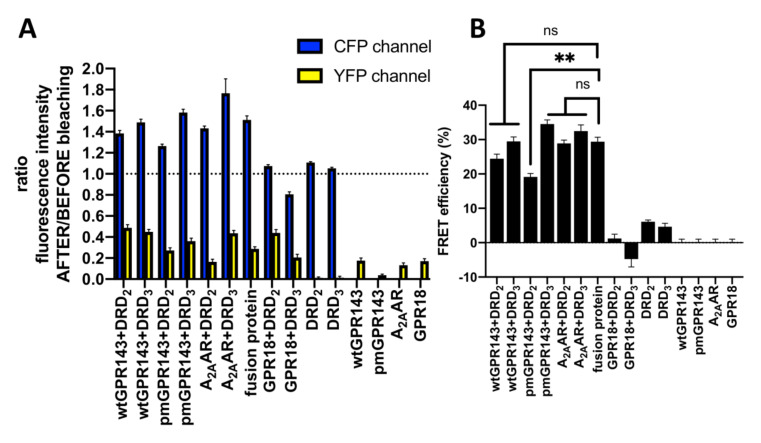
Quantification of acceptor photobleaching FRET. Wt or pmGPR143-YFP, A_2A_AR-YFP or GPR18-YFP and DRs-CFP (DRD_2_ or DRD_3_) were co-transfected in COS7s. (**A**) Ratio of emission intensity after:before bleaching was determined. Controls = single transfected COS7s, DRs-CFP + GPR18-YFP, and CFP-YFP fusion protein; (**B**) FRET efficiency was quantified for co-transfected COS7s. Controls = Single transfected cells, DRs-CFP + GPR18-YFP, and CFP-YFP fusion protein. Data represent means ± SEM of three independent experiments; on average, 92 ± 9 ROIs were analyzed per sample. Significant differences between controls (single transfected and +GPR18 samples) and treatment samples including the positive control CFP-YFP fusion protein were observed. Wt and pmGPR143 and A_2A_AR-transfected samples did not differ from the CFP-YFP fusion-protein, except for pmGPR143+DRD_2_. Values refer to limited regions (see Figures S3 and S4). * = *p* < 0.0001, ns = not significant.

To further validate GPR143 and DR interactions, we used a quantitative FRET approach, the acceptor photobleaching method. During FRET, the donor fluorescence is partially quenched by the acceptor. Photobleaching the acceptor irreversibly eliminates the quenching effect and the level of donor fluorescence increases. Thus, this method was used to measure donor “dequenching” as an indicator of colocalization. Bleaching was limited to designated regions of interest (ROI, Figure S2). For wtGPR143 samples, intracellular regions where colocalization was observed were chosen. Images captured before and after photobleaching display fluorescence in the CFP and YFP channels (Figure S2). The absolute fluorescence in the ROIs was used to calculate the ratio of emission intensity after versus before photobleaching and FRET efficiency (Figure 3). 

When GPR143 and DRs were coexpressed, the intensity of CFP emission increased, indicating that the two fluorophores were in close proximity and involved in resonance energy transfer before photobleaching (Figure 3). The FRET efficacy of the co-transfected receptors was slightly higher for DRD_3_ as compared to DRD_2_. While co-expression of wtGPR143 yielded similar FRET efficacies for both DRs (24.5 ± 1.3 for DRD_2_ and 29.5 ± 1.3 for DRD_3_), the co-expression of pmGPR143 with DRD_3_ (34.5 ± 1.2) was significantly higher compared to DRD_2_ (19.2 ± 1.0). When A_2A_AR was co-expressed with DRs, FRET efficacy reached up to 30% (28.9 ± 1.0 for DRD_2_ and 32.5 ± 1.8 for DRD_3_), which was comparable with the control pECFP-EYFP fusion protein (29.4 ± 1.3), suggesting maximal ratio. GPR18 was used as a negative control. Fluorescence ratios in the photobleaching experiment confirmed that, for GPR18, there was almost no CFP increase in fluorescence after photobleaching, indicating that the DRs do not interact with this receptor. The FRET efficacy calculated for the control experiment (1.2 ± 1.2 for DRD_2_ and −4.8 ± 2.3 for DRD_3_) was significantly different (*p* < 0.0001) from the positive controls (A_2A_AR-DRs and fusion protein CFP-YFP, Figure S3). In addition, the GPR18 transfected samples did not differ in FRET efficiency from the single transfected samples (*p* > 0.05). FRET efficiency values are similar to those reported in previous studies using CFP-YFP [1,45,46,47], suggesting that they can be considered reliable. Hence, we demonstrate that GPR143 and DRs directly interact with each other in several regions of the cell. 

### 2.2. GPR143 Influences Dopaminergic Signaling in the β-Arrestin Recruitment Assay

**Figure 4:**
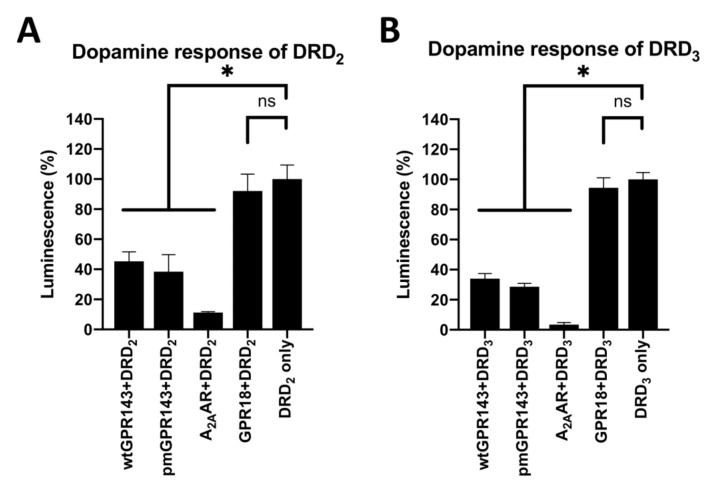
Dopamine response in CHO β-Arrestin cells expressing DRD_2_ and DRD_3_ co-transfected with a second GPCR. β-Arrestin assays were performed on dopamine treated CHO cells expressing DRD_2_ (**A**) or DRD_3_ (**B**), co-transfected with a second GPCR. The data were baseline corrected and correspond to 2–3 independent experiments performed in duplicates or triplicates. (**A**) Significant differences were observed between the DRD_2_ alone and the other dimer pairs (vs. wtGPR143+DRD_2_ *p* = 0.0113; vs. pmGPR143+DRD_2_ *p* = 0.0052 and vs. A_2A_AR+DRD_2_ *p* = 0.0052), except for GPR18+DRD_2_ where no significant difference was observed (*p* = 0.8082). (**B**) Significant differences were also observed between DRD_3_ alone and the other dimer pairs (* = *p* < 0.0001 for all), except for GPR18+DRD_3_ where no significant difference (ns = not significant) was observed (*p* = 0.1192).

Having determined that GPR143 and DRs, as well as A_2A_AR and DRs, also colocalize in CHO cells (Figure S4, panels A and B), we performed a β-Arrestin recruitment assay (PathHunter®, DiscoverX, Fremont, CA, USA) to determine if dimerization with GPR143 affects functionality of the DRs. This assay is based on enzyme fragment complementation of β-galactosidase. When a ligand binds and activates the GPCR, β-Arrestin-2 is recruited, thereby complementing the enzyme and rendering it active. The active β-galactosidase now can hydrolyze a substrate generating chemiluminescence, which can be quantitated as a correlate of receptor activation. Dopamine, the endogenous DR ligand chosen for the experiments, was used at 10 µM. A_2A_AR-DRD_2_-complex formation also modulates DRD_2_ ligand binding affinity and G-protein coupling [12] and A_2A_AR was therefore a suitable positive control. GPR18 was used as a negative control, as this receptor did not colocalize in FRET studies. Having established a functional system, we were able to determine a physiologically relevant functional interaction between GPR143 and DRs. Co-expression of either wt or pmGPR143 resulted in significant inhibition of the DR response towards dopamine (Figure 4, *p* = 0.0113 for wtGPR143+DRD_2_, *p* < 0.0001 for wtGPR143+DRD_3_, *p* = 0.0052 for pmGPR143+DRD_2_ and *p* < 0.0001 for pmGPR143+DRD_3_) compared to co-expression of A_2A_AR-DR which significantly inhibited the DRs response to dopamine at any concentration (up to 90%, Figure 4), in concordance with the antagonistic allosteric effect of the A_2A_AR on DRD_2_ and DRD_3_ [16,21,48,49,50]. Lastly, GPR18 did not have an effect on the DRs response to dopamine, as it was not different from the DR only controls. 

**Figure 5:**
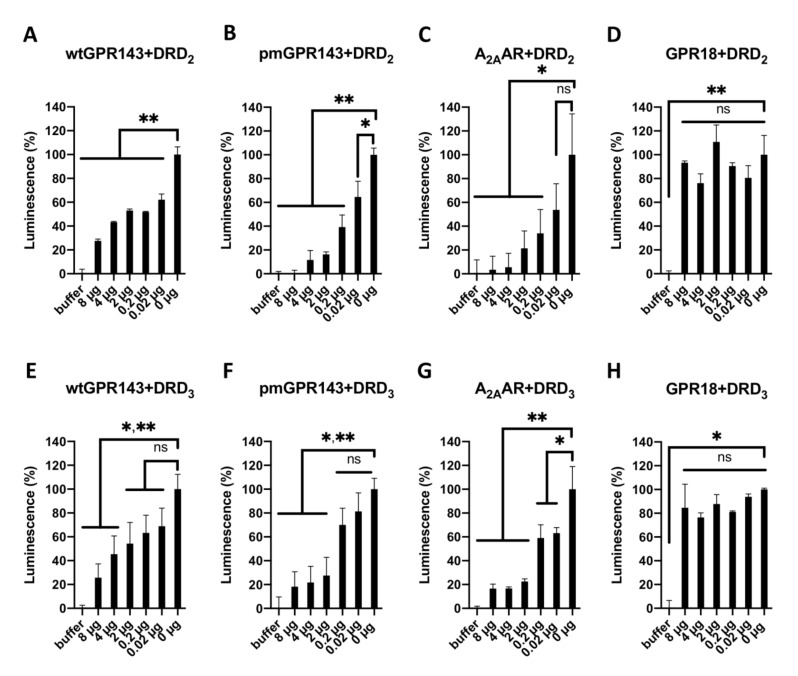
Dopamine response of CHO β-Arrestin DRD_2_ and DRD_3_ cells co-transfected with different concentrations of wtGPR143-, pmGPR143-, A_2A_AR- and GPR18-YFP in the β-Arrestin recruitment assay. CHO β-Arrestin DRD_2_ and DRD_3_ cells were transfected with different amounts of plasmids containing receptor cDNA (8, 4, 2, 0.2, and 0.02 µg) and treated with 10 µM dopamine (or buffer). Data were normalized to buffer (0%), and DRs only (0 µg, 100%) and correspond to mean ± SEM of two to three independent experiments performed in triplicates or quadruplicates. (**A**) Coexpression of wtGPR143 had a significant effect on DRD_2_ at all concentrations (*p* < 0.0001). (**B**) Coexpression of pmGPR143 had a significant effect overall on DRD_2_ activity (*p* < 0.0127). The 0 µg vs. 0.02 µg sample differed less (*p* = 0.003), while the other concentrations differed more significantly (*p* < 0.0001 for 0 µg vs. 0.2–8 µg). (**C**) Coexpression of A_2A_AR did not have a significant effect on DRD_2_ for 0 µg vs. 0.02 µg (*p* = 0.1037), while significant differences were observed for the other concentrations (0.2 µg *p* = 0.0445; vs. 2 µg *p* = 0.0222; vs. 4 µg *p* = 0.0066; vs. 8 µg *p* = 0.0066). (**D**) Coexpression of GPR18 had no significant effect on DRD_2_ (*p* > 0.05 for all). (**E**) Coexpression of wtGPR143 had a significant effect on DRD_3_ at concentrations of 4 µg and 8 µg (*p* = 0.0301, *p* = 0.0024). (**F**) Coexpression of pmGPR143 had a significant effect on DRD_3_ overall (*p* < 0.0001). Post-hoc comparisons indicated significant differences in activity for concentrations greater than 2 µg (vs. 2 µg *p* = 0.012; vs. 4 µg *p* = 0.0006, vs. 8 µg *p* = 0.0004). (**G**) Coexpression of A_2A_AR had a significant effect on DRD_3_. Post-hoc comparisons indicated that the transfection of 0.02 µg and 0.2 µg significantly differed from the 0 µg sample (*p* = 0.071; *p* = 0.0066, respectively), while higher concentrations had a greater effect (vs. 2–8 µg *p* < 0.0001 for all). (**H**) Coexpression of GPR18 had no significant effect on DRD_3_ (*p* > 0.05 for all, except for buffer). * = *p*≤ 0.05; ** = *p* ≤ 0.01. ns = not significant.

Next, we performed β-Arrestin assays with DRD_2_ or -D_3_ expressing cells co-expressing different amounts of GPR143 receptors (Figure 5) in order to show that changes in dopamine activation of DR’s correlated with the presence of GPR143 (or A_2A_AR). Cells stably transfected with DRs were transiently co-transfected with decreasing amounts of GPR143 or A_2A_AR plasmid (8, 4, 2, 0.2, 0.02 µg, for Western blot analysis confirmation, see Figure S5). The luminescence signal, corresponding to the DR activation by dopamine, was decreased with increasing amounts of co-transfected receptors. This was also the case for A_2A_AR, as expected [21]. We did not observe differences in the DR’s response towards dopamine for GPR18.

## 3. Discussion

DRs regulate numerous physiological functions and have been linked to numerous pathological conditions [7,8]. DRs have been shown to be very promiscuous and complex with many other GPCRs in order to carry out these multiple functions with specificity [7,14,16,19,51,52,53,54,55]. Among them, the A_2A_AR-DRD_2_-complex is the most studied heteromer, as it appears to be highly relevant for schizophrenia and Parkinson’s disease [3,7,56].

We previously demonstrated that pimozide, a DRD_2_ and DRD_3_ antagonist [23,24,25,26], also modulates activity of the orphan receptor GPR143 [22]. We therefore hypothesized that GPR143 and DRs may interact with each other in ocular and brain tissues [27,28]. Our hypothesis was supported by a number of studies that observed protein--protein interactions between GPR143 and other proteins, which similar to DRs, facilitate multiple functions. GRP143 binds MART-1 [36], tyrosinase (a key enzyme required for melanin synthesis) [1] and Gαi3 [38], which may underlie its regulation of melanosome differentiation and maturation. Furthermore, melanocytes express DRs 1–5 [57] and expression of DRs and GRP143 overlap in various areas of the brain [28].

Various roles have been proposed for GPR143; however, a precise function remains to be defined. Lack of GPR143 function results in formation of abnormal melanosomes [58]. Rather than small distinct organelles, macromelanosomes form, thus GPR143 appears to function as a “sensor” of melanosomal maturation to prevent formation of macro-organelles [30]. GPR143 function has also been shown to modulate the number of early-stage melanosomes [59] and form the trafficking fork separating lysosome and early melanosome bound proteins [60]. GPR143 may also control intracellular melanosome transport by regulating microtubule-mediated motility through interaction with tubulin [37]. Recent studies have shown that GPCRs can bind ligands on both membrane faces [61], which may underlie the interaction with tubulin. GPR143 may also regulate transcription of several melanosomal genes through modulation of the microphthalmia-associated transcription factor (MITF), thus forming a feedback loop being both a regulator and target of MITF [62,63]. In addition to its role in pigment cells, GPR143 has also been shown to mediate depressor response in the brain stem solitary nucleus [29], is expressed in several regions of the central nervous system such as the hippocampus [28], and, most recently, GPR143 was shown to be associated with nicotine addiction [64]. GPR143, like DRs, has multiple functions, numerous binding partners, and functions in various areas of the brain. 

In this study, we investigated the influence of GPR143 on DRD_2_ and DRD_3_. FRET studies demonstrated that GPR143 and DRs interact, while β-Arrestin recruitment assays showed that GPR143 can reduce DR activity by at least 50%. The reduction in activity may be due to reduced DR levels at the plasma membrane as we observed an increase in intracellular DRs colocalized with GPR143; alternatively, GPR143 may modulate DR affinity for dopamine or a combination of the two effects. 

The site of DR-GPR143 interaction appears to be determined by the location of GPR143, since we observed the difference depending on the GPR143 plasmid used for transfection. In pigment cells, wtGPR143 localizes intracellularly [40]. In non-pigment cells, such as COS cells or yeast (*Saccharomyces cerevisiae*), wtGPR143 is found in late endosomal/lysosomal fractions in the intracellular space or in the prevacuolar compartment, which are both functionally equivalent to melanosomes/late endosomes [1,41,42,43]. pmGPR143 sorts to the plasma membrane [1,22,65]. When wtGPR143 was co-transfected, DR-complexes were found exclusively in vesicles in the intracellular space and more often in the perinuclear region, while pmGPR143-DR-complexes were also found in vesicles in the intracellular space, but some appeared near the plasma membrane. It should, however, be noted that some studies reported that GPR143 protein can be found at the cell surface in human retinal pigment epithelial cells in situ but only 4% of total GPR143 protein [66], thus some interactions may take place at the plasma membrane. In addition, overexpression of GPR143 can cause accumulation at the cell surface [40,43]; however, our experiments were optimized such that we did not observe any accumulation at the plasma membrane [1,22]. 

The concept of orphan receptors modulating non-orphan receptors is not novel—for example, GPR50 modulates melatonin receptors MT1 and MT2 [67,68] and GPR143 itself has been shown to modulate the activity of the alpha-1B adrenergic receptor [39]. Consequently, GPR143 may play a similar role in modulating DR function. 

The results of the β-Arrestin assays demonstrated a significant reduction (at least 50%) in the DR response to activation with dopamine when GPR143 was co-expressed. The effect was less pronounced than the effect A_2A_AR had on the DRs (reduction of DR activity up to 90%), which is in concordance with previous studies that demonstrated that active A_2A_AR has a negative allosteric effect on dopaminergic signaling [11,21]. 

Although GPR143 is expressed primarily in pigment cells, several studies have suggested a link between the pigmentary system and dopaminergic signaling. For example, tyrosinase activity (which produces L-DOPA, a dopamine precursor, for pigment biosynthesis) and GPR143 expression are necessary for precise development of the optic tract [69], which may require an L-DOPA concentration gradient for correct nerve projection [70]. We have shown that GPR143 binds tyrosinase [1], which may allow for direct regulation of L-DOPA production and pigment synthesis. GPR143 may thus play a role in regulating both the production of L-DOPA by tyrosinase and dopamine-mediated signaling through DRs.

A potential model for DR-GPR143 interaction may involve dimer formation which occurs in late endosomes/multivesicular bodies, since these organelles are important for transport and sorting of proteins coming from the Golgi apparatus on their way to organelles such as lysosomes and melanosomes as well as for internalized receptors on their way to degradation or recycling [71]. It is also possible that the GPR143-DR interaction occurs during the transport of DRs to the plasma membrane. This interaction may, for example, delay DR recycling to the plasma membrane. While GPCR activity at the plasma membrane has been well characterized, recent studies have demonstrated a key role for intracellular activity of several GPCRs [72]; furthermore, this activity can be therapeutically targeted [73].

In conclusion, we have shown that GPR143 interacts with DRD_2_ and DRD_3_ and negatively modulates DR activity in response to dopamine. Furthermore, we have shown that GPR143-DR-complexes are primarily formed in vesicles in the intracellular space, even when a plasma membrane-localizing GPR143 variant, pmGPR143, was co-expressed. Whether GPR143 modulates DR activity by promoting its localization away from the plasma membrane and/or through allosteric modulation remains to be determined, as does the question of whether this mechanism is shared by melanocytes, the retinal pigment epithelium and other GPR143 expressing cells. 

## 4. Materials and Methods

### 4.1. Plasmids

The cDNA sequences of the human DRD2short and DRD3 genes were inserted into pE-CFP-N1 and pCMV-ARMS2-Prolink2 (DiscoverX, Fremont, CA, USA) vectors using NheI and HindIII restriction enzymes. For insertion into pCMV-ARMS2-Prolink2, an additional amino acid sequence (TC) was added to the 3′-end of the coding sequence. To generate pE-YFP-N1-wtGPR143, pE-YFP-N1-pmGPR143 and pE-YFP-N1-ADORA2A cDNA sequences of the human wtGPR143, pmGPR143 and ADORA2A were inserted into pE-YFP-N1 vector using KpnI and AgeI restriction enzymes as we previously described [1]. We removed the stop codon, so that the C terminal tag was in frame. pE-YFP-N1-GPR18 was kindly provided by the Mueller Lab (University of Bonn, Dept. Pharmaceutical and Medicinal Chemistry). The fusion protein pECFP-EYFP vector (referred to as CFP-YFP hereafter) was cloned as previously described ([1], supplemental methods file). CFP-YFP was used as internal control for statistical analysis. All plasmids were verified by sequencing (Eurofins Genomics Germany, Ebersberg, Germany). GPR143 is typically restricted to endosomal compartments in the intracellular space. It has been previously reported that the addition of a GFP tag at the C terminus does not affect the localization of GPR143 [30,32,42,43,74]. Therefore, the YFP protein was also fused to the C terminus.

### 4.2. Cell Culture and Transfection

Chinese Hamster Ovary (CHO) β-Arrestin cells were engineered by DiscoverX (Fremont, CA, USA) to express the β-galactosidase EA fragment fused to β-Arrestin. CHO β-Arrestin cells were cultured in a humidified incubator with 5% CO_2_ at 37 °C in Gibco F12 (Ham) (1×) medium supplemented with 10% FCS, 5 U/mL penicillin, 5 mg/mL streptomycin, 300 µg/mL hygromycin. β-Arrestin CHO cells were transfected with DRD_2_ and DRD_3_ expression plasmids using Lipofectamine™2000 (Thermo Fischer Scientific, Schwerte, Germany) according to the manufacturer’s recommendations and stable lines generated by antibiotic selection with G418. Cells were then maintained in 800 µg/mL G418 to maintenance of transfected cells. CHO β-Arrestin-DRD_2_ and -DRD_3_ cells were then transiently transfected with expression plasmids for wtGPR143, pmGPR143, and A_2A_AR or GPR18. COS7 cells were cultured in Dulbeccos’s modified eagle medium (DMEM) supplemented with 10 % fetal calf serum, 5 U/mL penicillin, and 5 mg/mL streptomycin at 37 °C with 10% CO_2_. Transient transfections were performed for microscopy experiments using Lipofectamine™ 2000 (Thermo Fischer Scientific, Schwerte, Germany) according to the manufacturer’s recommendations.

### 4.3. β-. Arrestin Assay

The β-Arrestin recruitment assay system PathHunter® developed by DiscoverX (Fremont, CA, USA, https://www.discoverx.com/arrestin, accessed on 30 July 2021) detects GPCR activation following ligand stimulation. The assay is based on enzyme fragment complementation of β-galactosidase. The assay is performed using a cell line expressing an Enzyme Acceptor (EA) which is fused to the β-Arrestin. The second part of the enzyme (ProLink/ PL) is fused to the C terminus of the GPCR of interest. EA and PL are inactive as single fragments. When a ligand binds and activates the GPCR of interest, the β-arrestin-2 protein is recruited to the GPCR since it is involved in receptor desensitizing and recycling. The recruitment of the β-arrestin leads to the complementation of the β-galactosidase. The active enzyme can catalyze hydrolysis and generate chemiluminescence when an appropriate substrate is proved. Thus, measured chemiluminescence (or β-galactosidase activity) correlates with receptor activation. In this study, the recruitment assay was performed using engineered CHO cell lines stably expressing the β-Arrestin protein linked to the EA fragment. The cell lines were transfected with the GPCR cDNA of interest fused to the ProLink-tag.

At 48 h post-transfection, co-transfected CHO β-Arrestin DRD_2_ and DRD_3_ cells were seeded in 96 well-plates (NunclonTM F96 MicroWellTM, Thermo Fischer Scientific, Scwerte, Germany). On the day of the assay, the medium was changed for 90 µl of F12 with 100 U/mL penicillin G, 100 µg/mL streptomycin, and the cells were incubated for at least 2 h at 37 °C with 5% CO_2_. Then, 10 µl of DMSO-diluted dopamine was added. Unsupplemented F12 medium was used as negative control. The final dimethyl sulfoxide (DMSO) concentration per well was 1%. After 90 min, the PathHunter® detection reagent (DiscoverX, Fremont, CA, USA) was added to the cell plate (50 µL/well) and incubated 1 h at room temperature in the dark, and finally chemiluminescence was detected by using the MikroWin2000 software and a multimode microplate reader (Mithras LB 940, Berthold Technologies, Bad Wildbad, Germany). 

### 4.4. Immunostaining

At 48 h post-transfection, the same co-transfected CHO β-Arrestin DRD_2_ and DRD_3_ cells used in the β-Arrestin recruitment assay were seeded on sterile coverslips in a 12 well-plate (Sarstedt, Nümbrecht, Germany) and cultured overnight. The next day (in parallel with the β-Arrestin assay), the CHO cells were fixed with 4% paraformaldehyde (pre-heated at 37 °C) for 20 min at room temperature. The cells were then washed with phosphate-buffered saline (1xPBS, pH 7.4) and blocked for 15 min with 1% bovine serum albumin (BSA)/1xPBS solution. Cells were incubated in the dark for 60 min with the primary antibody and then 30 min with the secondary antibody both diluted in 1% BSA/1xPBS. The primary antibody was diluted 1:1000 and the secondary antibody 1:500). 1xPBS was used to wash cells between and after antibody incubations. In addition, the nuclei of the cells were stained with DAPI (1 mg/µl diluted 1:1000 in 1% BSA/1xPBS) for 5 min in the dark. Finally, coverslips were mounted using Fluoromount™ Aqueous Mounting medium (Sigma-Aldrich, Merck KGaA, Darmstadt, Germany) and stored in the dark at 4 °C. The mouse monoclonal anti-PK/PL antibody (DiscoverX, Fremont, CA, USA) was used as the primary antibody and donkey anti-mouse-AlexaFluor594 (Jackson Immuno Research, Hamburg, Germany) as the secondary antibody to stain the Prolink tag on DRD_2_ and DRD_3_. Cotransfected receptors (wtGPR143, pmGPR143, and A_2A_AR) were tagged with a YFP-fluorophore-tag in order to reduce the spectral overlap of the fluorphores. A Nikon A1 Spectral confocal microscope operating with an argon laser (Pharmaceutical Institute, University of Bonn, Bonn, Germany) and the NIS Element Advanced Research software 4.0 were used for image acquisition and analysis. Each transfection and staining was repeated two to three times and at least ten squares (60× objective) containing 3–15 cells each were imaged for each sample. The cells that are most representative of the majority of each condition are shown.

### 4.5. Fluorescence Resonance Energy Transfer (FRET)

COS7 cells were seeded on sterile coverslips in 12 well-plates at 80–90% confluence and transfected with DRD_2_- and DRD_3_-CFP alone or in combination with YFP-tagged wtGPR143, pmGPR143 or A_2A_AR. After 48 h, cells were fixed with 4% paraformaldehyde (pre-heated at 37 °C) for 20 min at room temperature. The cells were then washed with phosphate-buffered saline (1xPBS, pH 7.4) and blocked for 15 min with 0.1% bovine serum albumin (BSA)/1xPBS solution. For the sensitized emission method, cells were mounted on slides using Fluoromount™ Aqueous Mounting medium (Sigma-Aldrich), while, for the photobleaching method, Mowiol 4–88 medium (Roth, Karlsruhe, Germany) was utilized as mounting medium. A Nikon A1 Spectral confocal microscope operating with an argon laser (Pharmaceutical Institute, University of Bonn, Bonn, Germany) and the NIS Element Advanced Research software 4.0 were used for image acquisition and analysis. Cells were examined with a 60× oil immersion objective. Each transfection and staining was repeated two to three times. The cell that was most representative of the majority of each condition was shown.

For the sensitized emission method, different optical configurations were set up: “Dd channel” for excitation and emission of the donor chromophore (ECFP, excitation filter: 457 nm, emission filter: 482/35 nm), “Aa channel” for excitation and emission of the acceptor chromophore (EYFP, excitation filter: 514 nm, emission filter: 540/30 nm) and “FRET channel” for the excitation of the donor and emission of the acceptor chromophore (excitation filter: 457 nm, emission filter: 540/30 nm). For each image, parameters (high voltage, offset, and laser intensity) were adjusted in order to limit the spectral bleed through and to avoid the pixel over-saturation. The laser intensity was equalized in both FRET and donor channel, while the high voltage was equalized in both FRET and acceptor channel. The FRET calibration was performed with single transfected cells expressing DRD_2_-CFP, DRD_3_-CFP, A_2A_AR -YFP, wtGPR143-YFP, pmGPR143-YFP, or GPR18-YFP. Correction parameters (CoA and CoB) were calculated by the software using the following formulas:

CoA = Da ACCEPTOR/Aa ACCEPTOR(1)

CoB = Da DONOR/Dd DONOR,(2)

where Aa corresponds to the channel where the excitation and the emission of acceptor is measured, Dd corresponds to the channel of excitation and emission of the donor, and Da corresponds to the channel of the excitation of the donor and emission of the acceptor. Xx DONOR/ACCEPTOR coefficients are average intensities of the donor/acceptor-only images. The corrected FRET signal and the FRET efficiency were calculated for each image using the following formulas:

FRET_CORR_= Da FRET ‒ (Dd FRET x CoB) ‒ (Aa FRET × CoA) (3)

FRET EFFICIENCY [%]= (FRET CORR / Dd FRET) × 100(4)

where Xx FRET members are average intensities of assigned FRET image components.

For the acceptor photobleaching method, images were captured before and after the photobleaching of acceptor molecules in a specific region of the cell (ROIs). If any interactions leading to energy transfer were present, photobleaching of the acceptor will lead to an increase of donor fluorescence, as it is no longer quenched by the acceptor. Acceptor photobleaching was performed with a high-intensity laser pulse at 514 nm. Images in the Aa and Dd channels were captured simultaneously before and after the photobleaching. FRET efficiency was calculated using the following formula:

FRET EFFICIENCY [%] = (IA ‒ IB) × 100/ IA(5)

where IA is the CFP intensity emission after bleaching, and IB is the CFP intensity emission before bleaching.

### 4.6. Western Blot

At 48 h post-transfection, co-transfected CHO β-Arrestin DRD_2_ and DRD_3_ expressing cells were scrapped of the plates, washed with PBS, and lysed on ice using an ultra-sound sonicator. The protein concentration was determined using a Bradford reagent. In addition, 30 µg of sample protein was mixed with loading buffer containing 2% SDS and warmed at 37 °C for 30 min. Then, proteins were separated on 10% SDS-PAGE gel and then transferred to a nitrocellulose membrane (PROTRAN—Nitrocellulose Transfer Membrane—Whatman, Sigma, Taufkirchen, Germany). The Mark12™ Unstained Standard and PageRuler™ Prestained Protein Ladder were used as protein markers. The membrane was blocked 1 h in a 5% powdered milk/PBS-Tween solution. Afterwards, the membrane was incubated with the primary antibody overnight at 4 °C or 1 h at RT, washed 1 h with PBS-Tween, incubated with secondary antibody and washed again. The detection was performed with ECL kit (GE Healthcare, Amersham, Arlington, IL, USA—Dassel, Germany) according to the manufacturer’s instructions. The following antibodies were used: mouse monoclonal anti-GFP (Biolegend, San Diego, CA, USA) and anti-mouse-HRP (Jackson Immuno Research, Hamburg, Germany).

### 4.7. Data Analysis

Data were analyzed using Prism 8.0 (GraphPad Software Inc., San Diego, CA, USA). NIS Element Advanced Research software 4.0 was used for microscopy image acquisition and analysis.

### 4.8. Statistical Analysis

Ordinary one-way ANOVA and the Holm–Sidak test for post-hoc comparisons were used for statistical analysis of the data.

## Data Availability

We did not report any data.

## Supplementary Materials

The following are available online at https://www.mdpi.com/article/10.3390/ijms22158328/s1, Figure S1: Control images of sensitized emission FRET; Figure S2: Acceptor photobleaching FRET in COS7 cells; Figure S3: Control images of FRET acceptor photobleaching.; Figure S4: Colocalization of GPR143 and DRs by immunofluorescence in CHO cells; Figure S5: Western blot analysis shown as an example for wtGPR143+DRD3.
