# Supplementary material for: Evidence for Protein–Protein Interaction between Dopamine Receptors and the G Protein-Coupled Receptor 143
Source: Int J Mol Sci. 2021 Aug 3;22(15):8328. doi: 10.3390/ijms22158328 (PMC8348196; doi:10.3390/ijms22158328)
Supplement: Supplementary file 1 [file ijms-22-08328-s001.zip › ijms-1294663-supplementary.pdf]

International Journal of *Molecular Sciences*

MDPI

Article

Evidence for Protein–Protein Interaction between Dopamine Receptors and the G Protein-Coupled Receptor 143

Beatriz Bueschbell [1,2], Prashiela Manga [3], Erika Penner [4] and Anke C. Schiedel [4,*]

1 Center for Neuroscience and Cell Biology, University of Coimbra, 3004-504 Coimbra, Portugal; btrb@uni-bonn.de (B.B.)
2 PhD Programme in Experimental Biology and Biomedicine, Institute for Interdisciplinary Research (IIIUC), University of Coimbra, Casa Costa Alemão, 3030-789 Coimbra, Portugal; btrb@student.uc.pt (B.B.)
3 Ronald O. Perelman Department of Dermatology, Grossman School of Medicine, New York University, New York, NY, USA; Prashiela.Manga@nyulangone.org (P.M.)
4 Department of Pharmaceutical & Medicinal Chemistry, Pharmaceutical Institute, University of Bonn, D-53121 Bonn, Germany; erikapenner@hotmail.de (E.P.), schiedel@uni-bonn.de (A.C.S.)
* Correspondence: Anke C. Schiedel, Department of Pharmaceutical & Medicinal Chemistry, Pharmaceutical Institute, University of Bonn, D-53121 Bonn, Germany; D-53121 Bonn, Germany; schiedel@uni-bonn.de.

Supplemental information

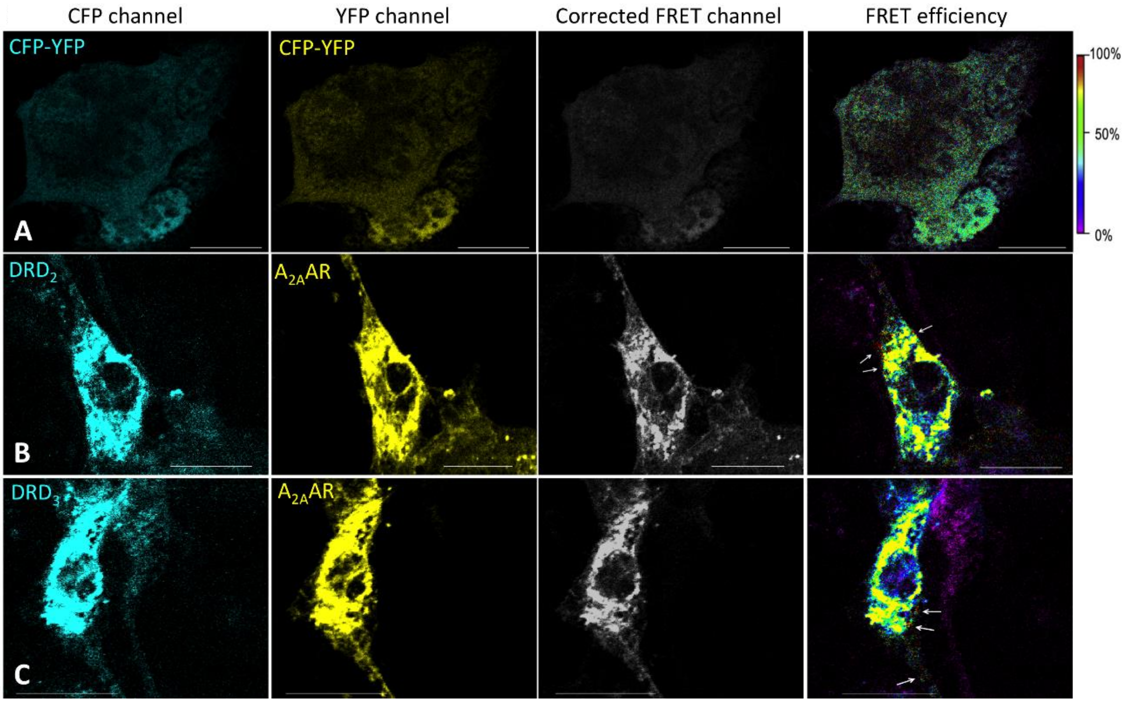

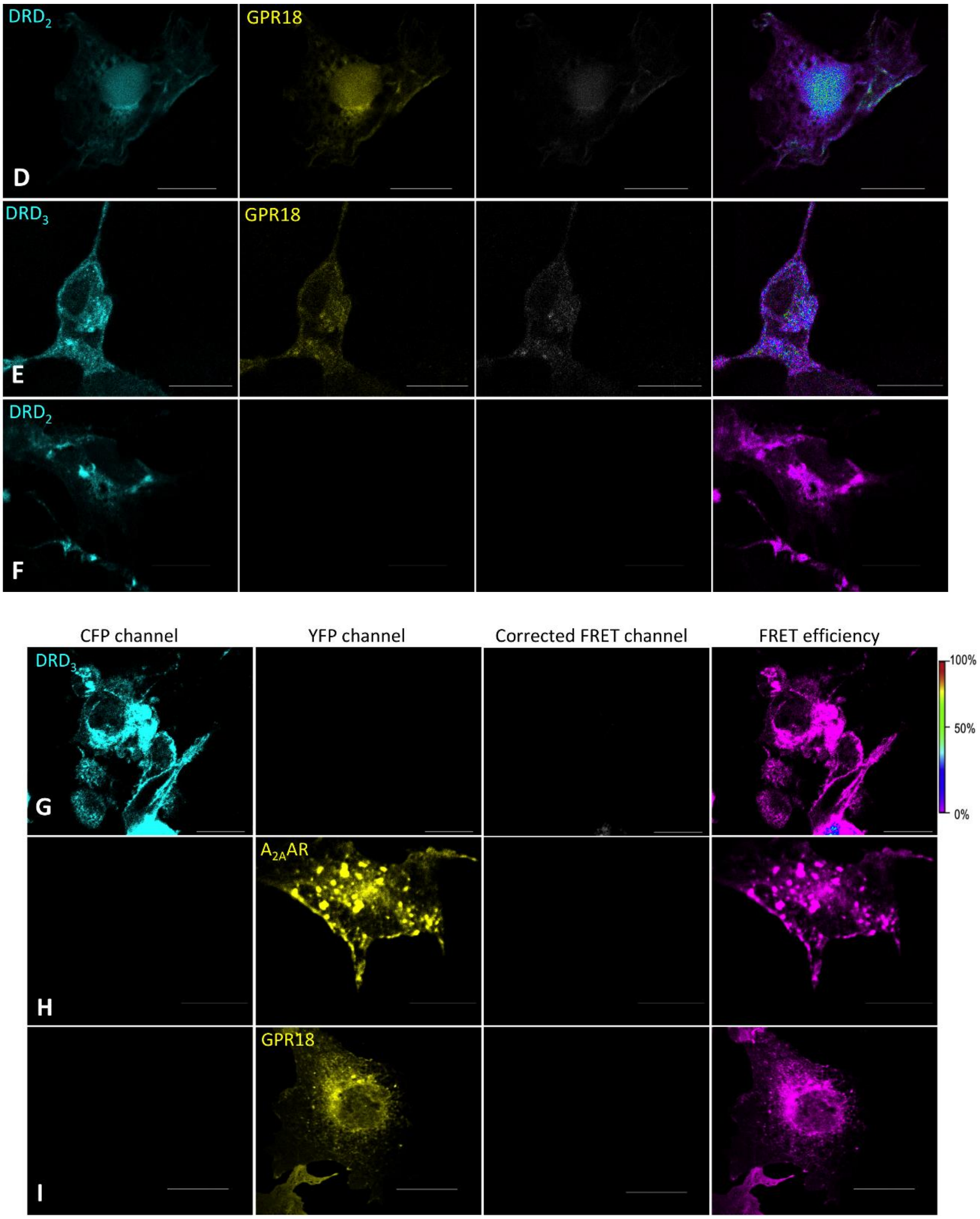

DRD2
GPR18
D
DRD3
GPR18
E
DRD2
F
CFP channel
YFP channel
Corrected FRET channel
FRET efficiency
DRD3
G
100%
50%
0%
A2AAR
H
GPR18
I

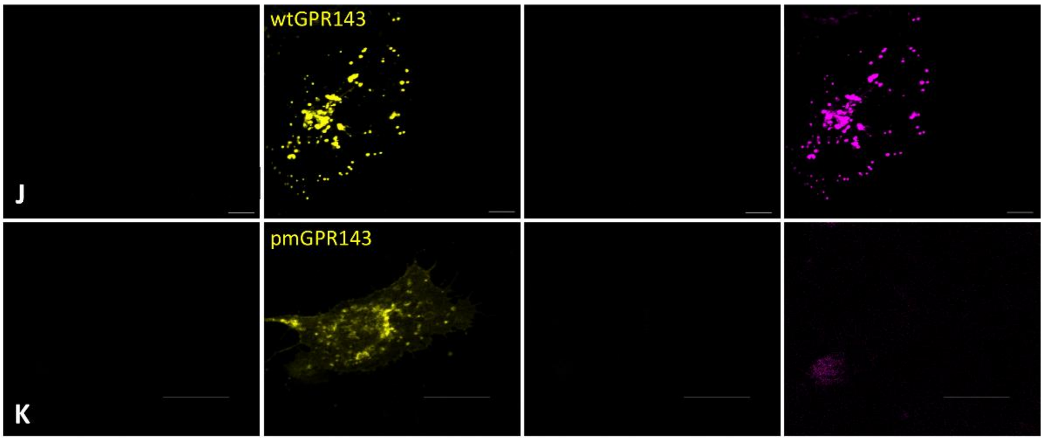

Figure S1. Control images of sensitized emission FRET. Sensitized emission method was used to detect interaction of GPR143 (YFP channel) and DRs (CFP channel) in COS7s transfected either with (**A**) CFP-YFP fusion protein, (**B**) DRD2-CFP and A2AAR-YFP, (**C**) DRD3-CFP and A2AAR-YFP as positive controls, (**D**) DRD2-CFP and GPR18-YFP, (**E**) DRD3-CFP and GPR18-YFP as negative controls (**F**) DRD2-CFP, (**G**) DRD3-CFP, (**H**) A2AAR-YFP, (**I**) GPR18-YFP, (**J**) wtGPR143-YFP or (**K**) pmGPR143-YFP. FRET signal, corrected by CoA and CoB parameters, and FRET efficiency (color scale on the far right)are shown. Scale bar = 20 µm. FRET, fluorescence resonance energy transfer; wt, wildtype, pm, plasma membrane.

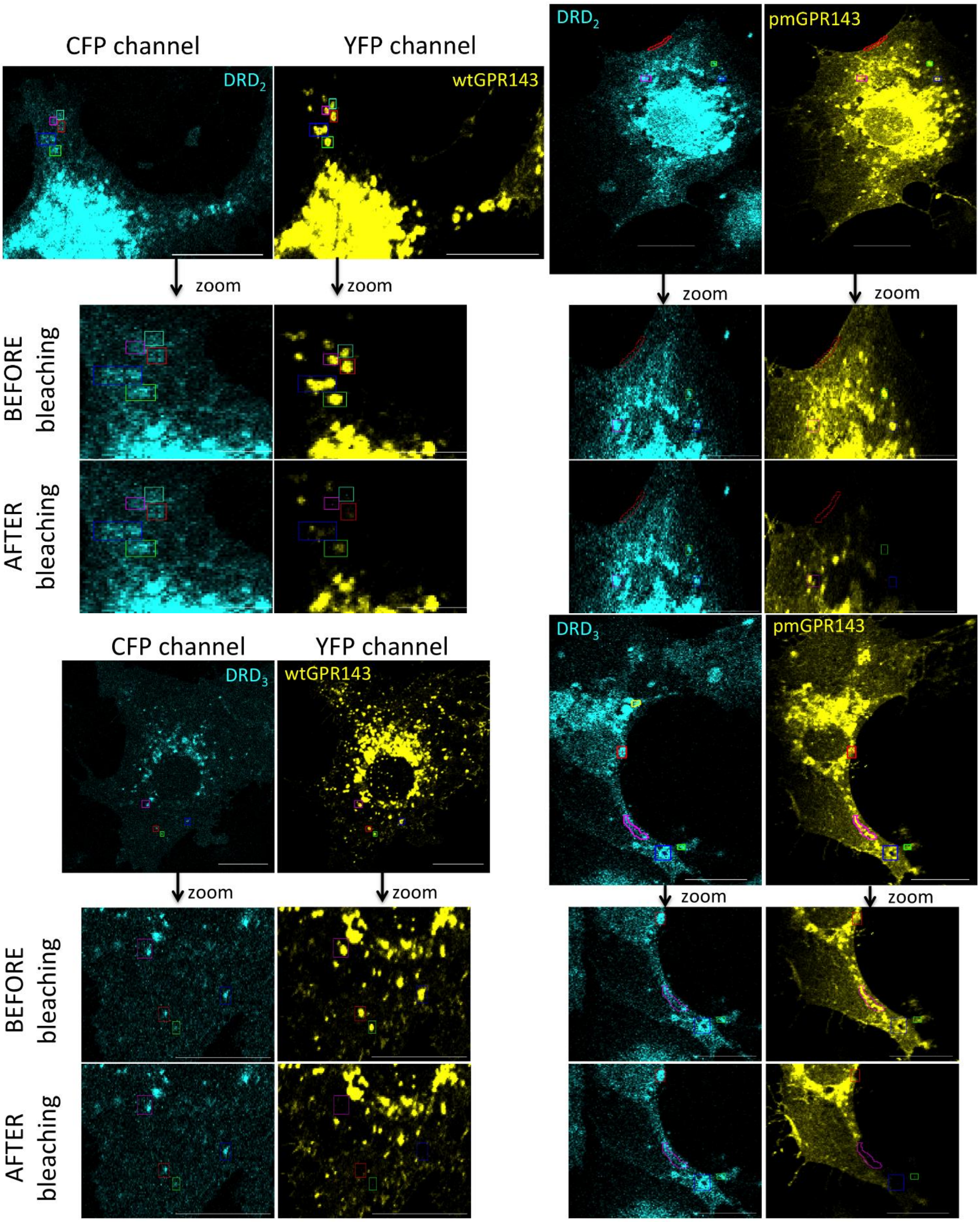
CFP channel
YFP channel
DRD$_2$
wtGPR143
zoom
zoom
BEFORE bleaching
AFTER bleaching
DRD$_2$
pmGPR143
zoom
zoom
CFP channel
YFP channel
DRD$_3$
wtGPR143
zoom
zoom
BEFORE bleaching
AFTER bleaching
DRD$_3$
pmGPR143
zoom
zoom

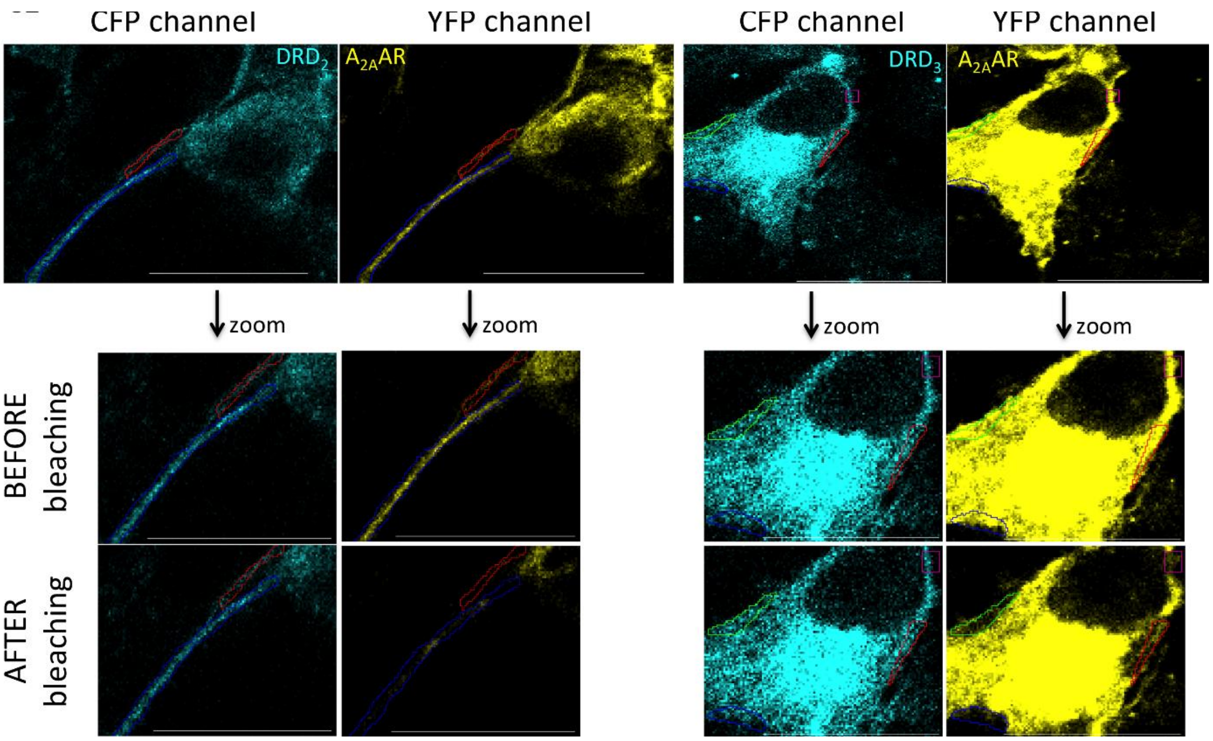

Figure S2. Acceptor photobleaching FRET in COS7 cells. COS7s were co-transfected with wt or pmGPR143-YFP or A_{2A}AR-YFP and DRs-CFP. The YFP photobleaching was performed and detected in delimited regions (highlighted and zoomed in the pictures). The fluorescence of GPR143 (YFP channel) and DRs (DRD_2 or DRD_3; CFP channel) were detected before and immediately after the acceptor photobleaching. Controls are shown in Figure S5. Scale bar = 20 μm.

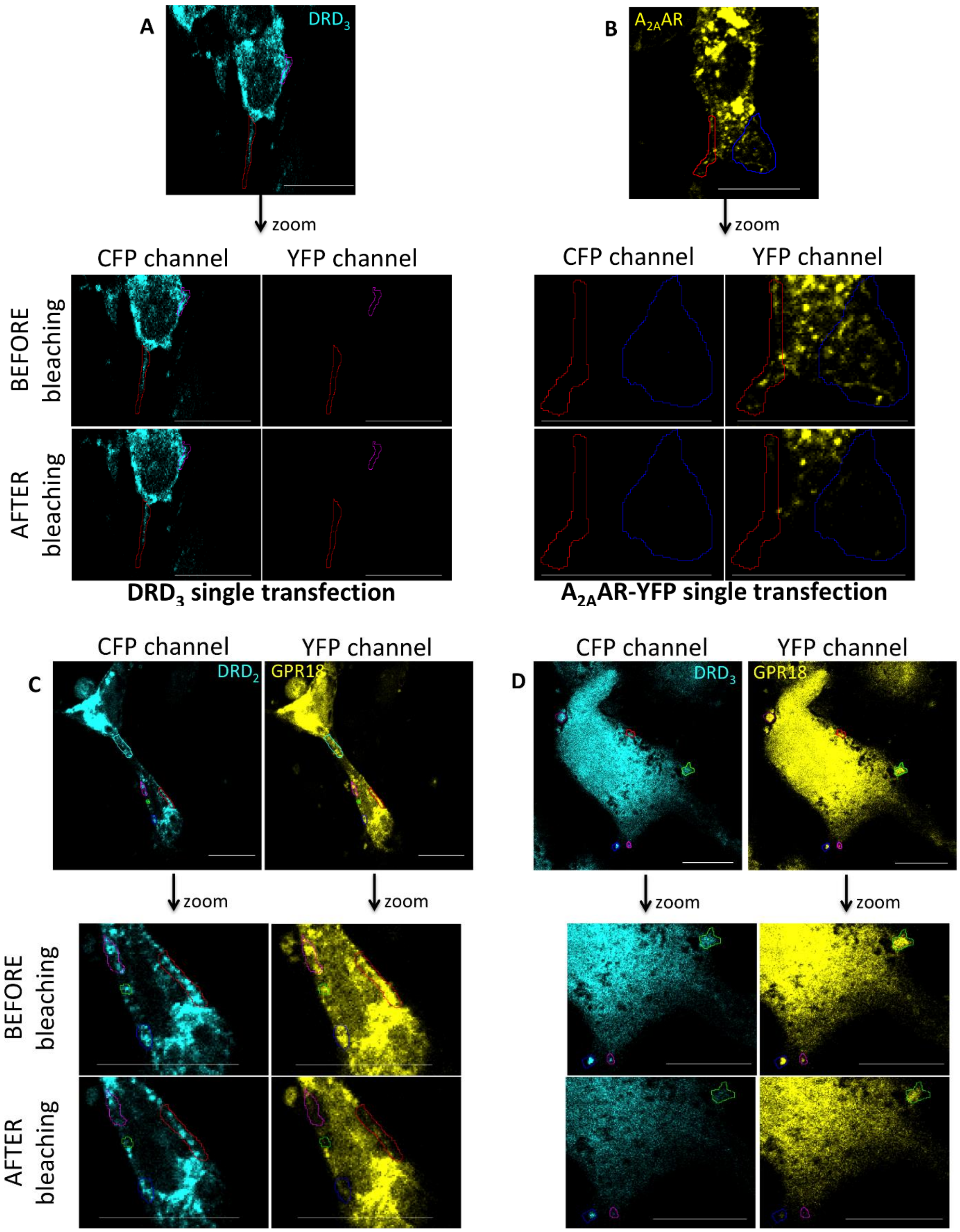
A
DRD3
zoom
CFP channel
YFP channel
BEFORE bleaching
AFTER bleaching
DRD3 single transfection
B
A2AAR
zoom
CFP channel
YFP channel
A2AAR-YFP single transfection
C
CFP channel
YFP channel
DRD2
GPR18
zoom
zoom
BEFORE bleaching
AFTER bleaching
D
CFP channel
YFP channel
DRD3
GPR18
zoom
zoom

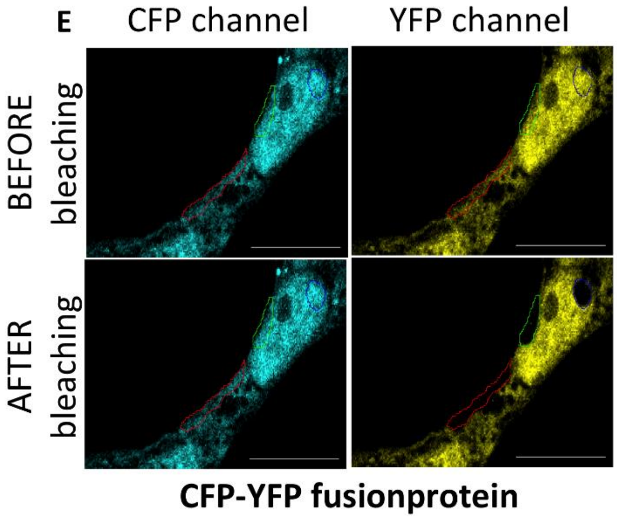

Figure S3. Control images of FRET acceptor photobleaching. The YFP photobleaching was performed and detected in delimited regions (highlighted and zoomed in the pictures) of COS7s transfected with either DRs-CFP, here DRD_3 as representative (**A**) or GPCR-YFP (**B**), here $A_{2A}AR$ as representative. CFP-YFP was used as positive control (E), while co-transfections with GPR18 (**C**, **D**) were used as negative controls. Images of all sample fluorescence are shown before and immediately after the acceptor photobleaching for CFP and YFP channels. For corresponding FRET efficacies see Figure 3. Scale bar = 20 µm.

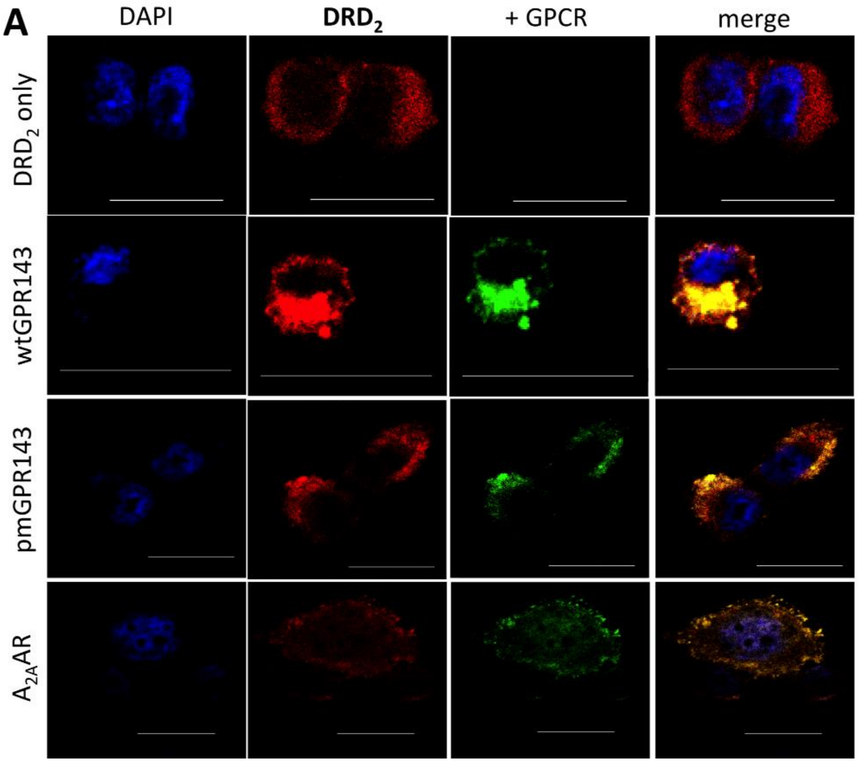

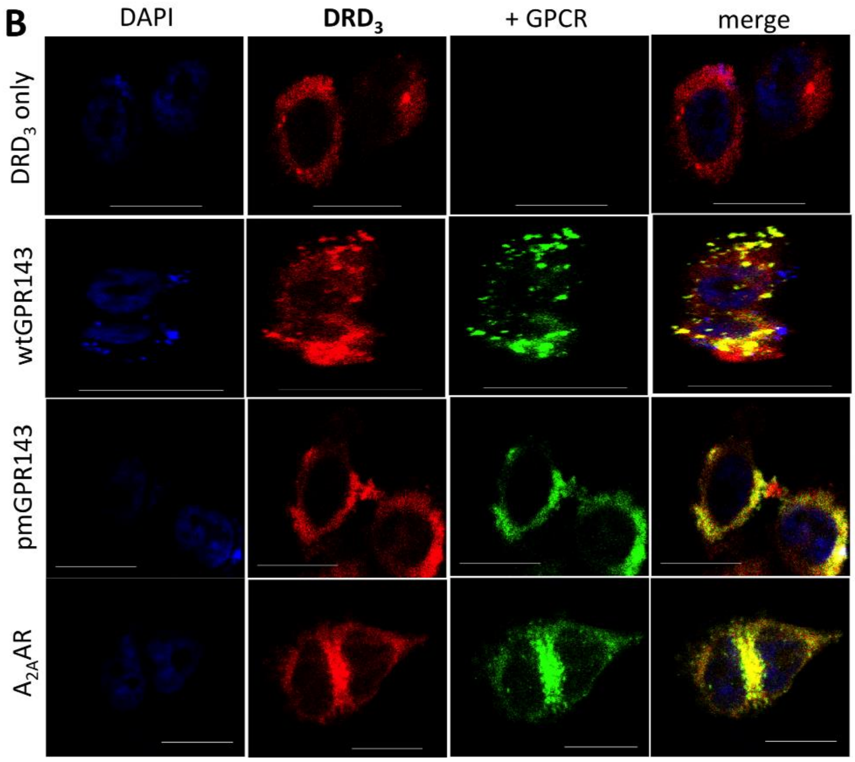

Figure S4. Colocalization of GPR143 and DRs by immunofluorescence in CHO cells. A: CHO DRD_2 cells were co-transfected with wt or pmGPR143-YFP, or $A_{2A}AR$ -YFP, fixed and stained with anti-ProLink (against PL tagged-DRD_2) and DAPI (nuclei). AlexaFluor594 was used as secondary antibody. **B:** CHO DRD_3 cells were co-transfected with wt or pmGPR143-YFP, or $A_{2A}AR$-YFP, fixed and stained with anti-ProLink (against PL tagged-DRD_3) and DAPI (nuclei). AlexaFluor594 was used as secondary antibody. DR only samples are shown in the first row of A and B**.** Scale bar = 20 μm.

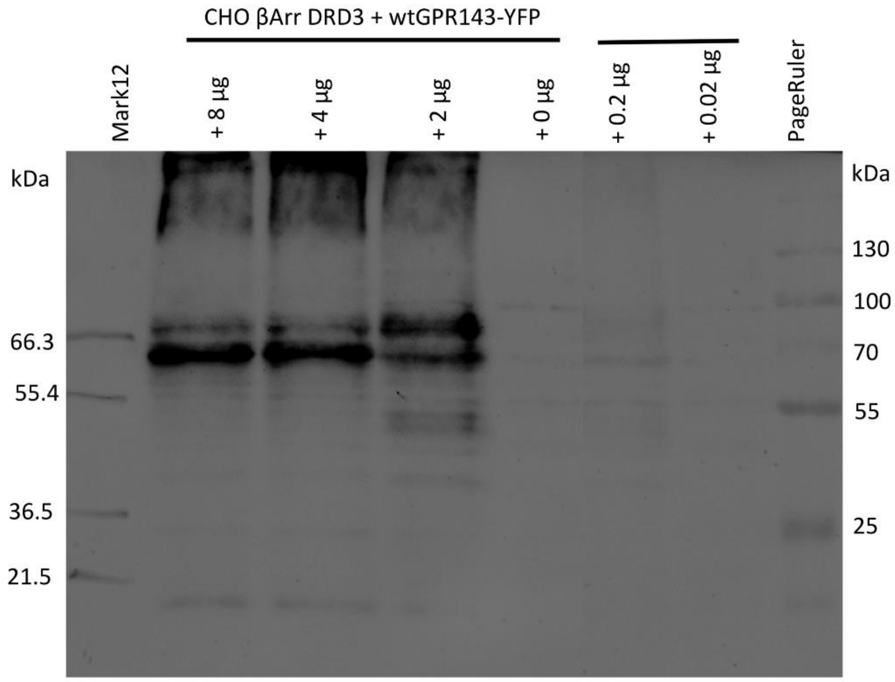

Figure S5. Western Blot Analysis shown as example for wtGPR143+DRD_3. CHO β-Arrestin cells stable expressing DRD_2 or DRD_3 were co-transfected with wt or pmGPR143-YFP, $A_{2A}AR$-YFP or GPR18-YFP in different concentrations (8, 4, 2, 0.2 or 0.02 μg). Untransfected CHO β-Arrestin cells

were used as control. 30 μg lysates were separated in 10% SDS-PAGE and transferred onto nitrocellulose. The anti-GFP tag (mouse monoclonal) antibody was used as primary and rabbit anti-mouse horseradish peroxidase–coupled antibody was used as secondary antibody, both in 1:5000 dilutions.
